# Integrated Transcriptome Landscape of mRNAs, lncRNAs, circRNAs, and miRNAs Reveals Molecular Regulatory Networks of Sex Differentiation in the Zig-Zag Eel (*Mastacembelus armatus*)

**DOI:** 10.3390/ijms27115111

**Published:** 2026-06-05

**Authors:** Junxian Zhu, Xianghui Jia, Liqin Ji, Chen Chen, Caixia Gao, Xiaoyou Hong, Xiaoli Liu, Chengqing Wei, Xinping Zhu, Wei Li

**Affiliations:** 1 Key Laboratory of Tropical and Subtropical Fishery Resources Application and Cultivation, Ministry of Agriculture and Rural Affairs, Pearl River Fisheries Research Institute, Chinese Academy of Fishery Sciences, Guangzhou 510380, China; zjzhujunxian@prfri.ac.cn (J.Z.); 15265228269@163.com (X.J.); jiliqin@prfri.ac.cn (L.J.); chenchen@prfri.ac.cn (C.C.); hxy@prfri.ac.cn (X.H.); liuxl@prfri.ac.cn (X.L.); zjweichengqing@prfri.ac.cn (C.W.); zhuxinping@prfri.ac.cn (X.Z.); 2 Guangdong Provincial Laboratory Animals Monitoring Center (Guangdong Provincial Biotechnology Research Institute), Guangzhou 510663, China; gcx@gdlami.com

**Keywords:** *Mastacembelus armatus*, sex differentiation, sexual dimorphism in growth, transcriptome, non-coding RNAs

## Abstract

The zig-zag eel (*Mastacembelus armatus*) exhibits sexual dimorphism in growth patterns. Identifying the genes involved in sex differentiation is a crucial step toward achieving single-sex breeding and serves as a vital foundation for elucidating the XY sex determination mechanism in *M. armatus*. This study measured the morphological characteristics of male and female *M. armatus* and found that males were significantly superior to females in body weight and nearly all morphological indices. Subsequently, whole-transcriptome sequencing was performed on the gonads of adult males and females, identifying 11,714 DEmRNAs, 3442 DElncRNAs, 416 DEcircRNAs, and 620 DEmiRNAs, including male sex differentiation genes such as *Sox30*, *Tbx1*, *Sox9*, and *Gata4*, and female sex differentiation genes like *Sox3*, *Foxl2*, and *Wnt4a*. Functional enrichment analysis identified pathways associated with sex differentiation, including the TGF-beta signaling pathway, the steroid hormone biosynthesis, the Hippo signaling pathway, and the Wnt signaling pathway, etc. A ceRNA network was constructed based on differentially expressed mRNAs and ncRNAs, revealing that the sex differentiation-related genes *Sox3*, *Sox9*, *Sox30*, *Tbx1*, and *Wt1* are regulated by one or multiple pairs of lncRNA/circRNA-miRNA pairs. The study results will provide molecular targets for research on sex-controlled breeding in *M. armatus* and lay an important theoretical foundation for clarifying its sex differentiation mechanisms.

## 1. Introduction

Sexual dimorphism is a widespread phenomenon in the natural world, characterized by significant differences between male and female individuals in morphology [1,2], physiology [3,4], behavior [5,6], and growth performance [7,8]. Sexual dimorphism in growth traits holds significant economic importance in aquaculture, and elucidating its regulatory mechanisms is crucial for achieving sex-controlled breeding and enhancing farming yields and profitability [9,10]. The zig-zag eel (*Mastacembelus armatus*) is a premium freshwater fish species with considerable commercial value, which exhibits marked sexual dimorphism in growth patterns, with males typically growing faster than females [11,12]. Consequently, there exists an industrial demand for all-male breeding of *M. armatus*. Identifying key genes involved in sex differentiation and unraveling their regulatory network is a key step toward achieving sex-controlled breeding [13,14]. Currently, systematic analyses of key genes associated with sex differentiation and their regulatory networks in *M. armatus* remain relatively limited.

Moreover, regulation of sex differentiation is a complex network composed of multiple types of molecules, extending beyond the mRNA level [15]. Although previous studies have confirmed that sex-determining genes such as *Dmrt1* [16], *Gsdf* [17], and *Foxl2* [18] play critical roles in sexual differentiation, research also suggests that the non-coding RNAs (ncRNAs) may hold potential value in this process [19,20]. However, studies on sex-related ncRNAs in *M. armatus* are still relatively scarce. Therefore, systematic analysis of the sexually dimorphic expression profiles and their interactive relationships of mRNAs, lncRNAs, circRNAs, and miRNAs in *M. armatus* provides a critical entry point for identifying key molecules driving sex differentiation and clarifying the regulatory mechanisms underlying its sexually dimorphic growth, potentially offering key molecular targets for sex-controlled breeding in *M. armatus*.

This study employed high-throughput transcriptome sequencing of male and female gonads in *M. armatus* to identify sexually dimorphic expression profiles of mRNAs, lncRNAs, circRNAs, and miRNAs and screen key differentially expressed molecules and pathways associated with sex differentiation. Meanwhile, a ceRNA (competing endogenous RNA) regulatory network was constructed to elucidate the transcriptional regulatory relationships among sexually dimorphic expression molecules. Our findings provide a molecular genetic foundation for investigating the sex differentiation mechanisms in *M. armatus* and offer a potential molecular target for sex-controlled breeding.

## 2. Results

### 2.1. Sexual Dimorphism in Growth Patterns Presents in M. armatus

**Figure 1:**
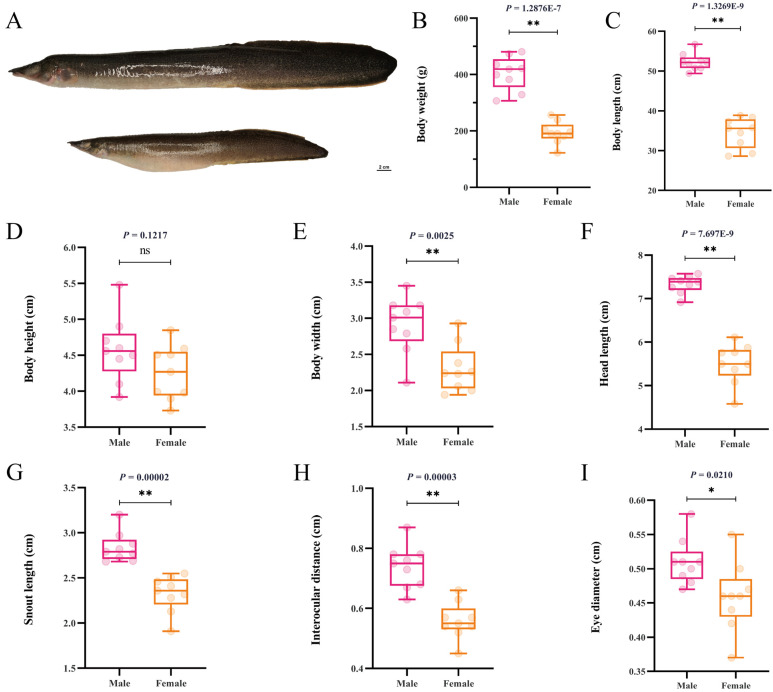
Morphological comparison between male and female *M. armatus*. (**A**) Representative images of collected male and female *M. armatus*. Comparative analyses of (**B**) body weight, (**C**) body length, (**D**) body height, (**E**) body width, (**F**) head length, (**G**) snout length, (**H**) interocular distance, and (**I**) eye diameter in both sexes. The box line represents the median, and the whiskers indicate the maximum and minimum values. The different colored bubbles represent each individual sample in groups. *, *p* < 0.05; **, *p* < 0.01; ns, not significant.

Morphological measurements of sampled *M. armatus* revealed significant differences in growth patterns (body weight and size) between males and females of the same age (Figure 1A). Male individuals significantly exceed female individuals in body weight (Figure 1B), body length (Figure 1C), body width (Figure 1E), head length (Figure 1F), snout length (Figure 1G), interocular distance (Figure 1H), and eye diameter (Figure 1I) (*p* < 0.05). There is no significant difference in body height between males and females (Figure 1D).

### 2.2. Identification and Characterization of Differentially Expressed mRNAs

**Figure 2:**
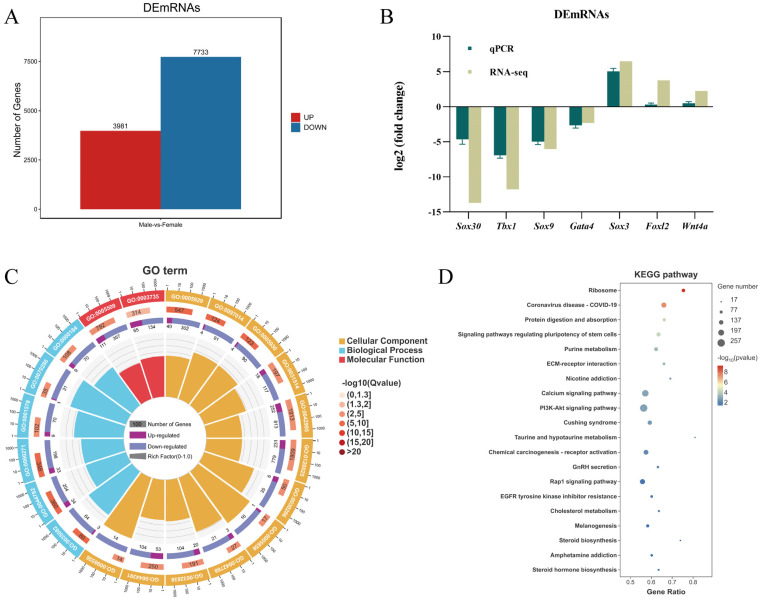
Analysis of mRNA profiles in male and female *M. armatus* gonads. (**A**) Identifying DEmRNAs between male and female gonads. (**B**) The qPCR validation of seven randomly selected sex differentiation-related genes and qPCR data are presented as mean ± standard deviation. (**C**) GO enrichment analysis of DEmRNAs, displaying top 20 significantly enriched GO terms. (**D**) KEGG enrichment analysis of DEmRNAs, displaying top 20 significantly enriched KEGG pathways.

Subsequently, whole-transcriptome sequencing was performed using gonadal tissues collected from males and females of *M. armatus*. RNA sequencing (RNA-seq) yielded 12,485,392,200 to 13,999,079,000 clean reads, with Q20 values ranging from 97.34 to 97.79%, Q30 values ranging from 92.80 to 93.78%, and GC content ranging from 46.80 to 48.24% (Table S1). Principal component analysis (PCA) and correlation analysis results indicated that samples within the same gonad group exhibited high similarity, while distinct differences were observed between the two groups (Figure S1), suggesting a sexually dimorphic expression profile between male and female gonads of *M. armatus*. A total of 11,714 differentially expressed mRNAs (DEmRNAs) were identified in the gonads of male and female *M. armatus*, of which 3981 were upregulated and 7733 were downregulated (Figure 2A) (Table S2). Seven genes associated with sex differentiation (*Sox30*, *Tbx1*, *Sox9*, *Gata4*, *Sox3*, *Foxl2*, and *Wnt4a*) were randomly selected for quantitative real-time PCR (qPCR) validation. The results were consistent with the transcriptome sequencing data (Figure 2B), indicating the reliability of the RNA-seq findings. The GO (Gene Ontology) and KEGG (Kyoto Encyclopedia of Genes and Genomes) analysis revealed that DEmRNAs were significantly enriched in 42 GO terms and 41 KEGG pathways (Figure 2C,D) (Table S2), with pathways related to sexual differentiation including the PI3K-Akt signaling pathway, the estrogen signaling pathway, the TGF-beta signaling pathway, and so on.

### 2.3. Identification and Characterization of Differentially Expressed lncRNAs

**Figure 3:**
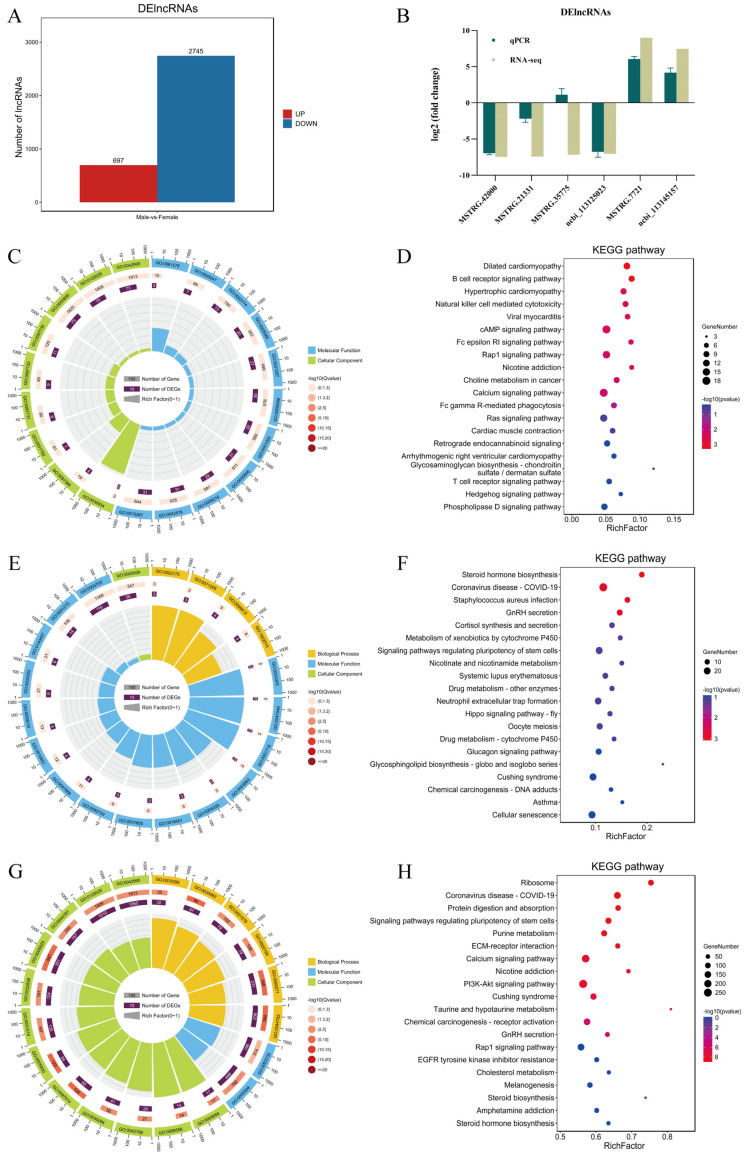
Analysis of lncRNA profiles in male and female *M. armatus* gonads. (**A**) Identifying DElncRNAs between male and female gonads. (**B**) The qPCR validation of six randomly selected DElncRNAs and qPCR data are presented as mean ± standard deviation. GO enrichment analysis of genes affected by DElncRNAs via antisense (**C**), cis-acting (**E**), and trans-acting (**G**) regulatory mechanisms, showing top 20 enriched GO terms. KEGG enrichment analysis of genes affected by DElncRNAs via antisense (**D**), cis-acting (**F**), and trans-acting (**H**) regulatory mechanisms, showing top 20 enriched KEGG pathways.

Meanwhile, 3442 differentially expressed lncRNAs (DElncRNAs) were identified in the RNA-seq data, comprising 697 upregulated and 2745 downregulated (Figure 3A) (Table S3). Six DElncRNAs were randomly selected for qPCR validation, and the qPCR results matched the transcriptome data well (Figure 3B), confirming the robustness of the RNA-seq analysis. LncRNAs can regulate gene transcription through antisense, cis-acting, and trans-acting mechanisms [21]. Therefore, functional enrichment analysis was performed on genes affected by DElncRNAs (Table S3). Results revealed that in antisense regulation, affected genes were enriched in 6878 GO terms, and 32 KEGG pathways showed significant enrichment (Figure 3C,D). In cis-acting regulation, the affected genes were enriched in 10,437 GO terms, and 16 KEGG pathways were significantly enriched (Figure 3E,F). In trans-acting regulation, the affected genes were significantly enriched in 43 GO terms and 41 KEGG pathways (Figure 3G,H). Collectively, terms and pathways associated with sex differentiation contained the GnRH secretion, the cortisol synthesis and secretion, the steroid hormone biosynthesis, and so on.

### 2.4. Identification and Characterization of Differentially Expressed circRNAs

**Figure 4:**
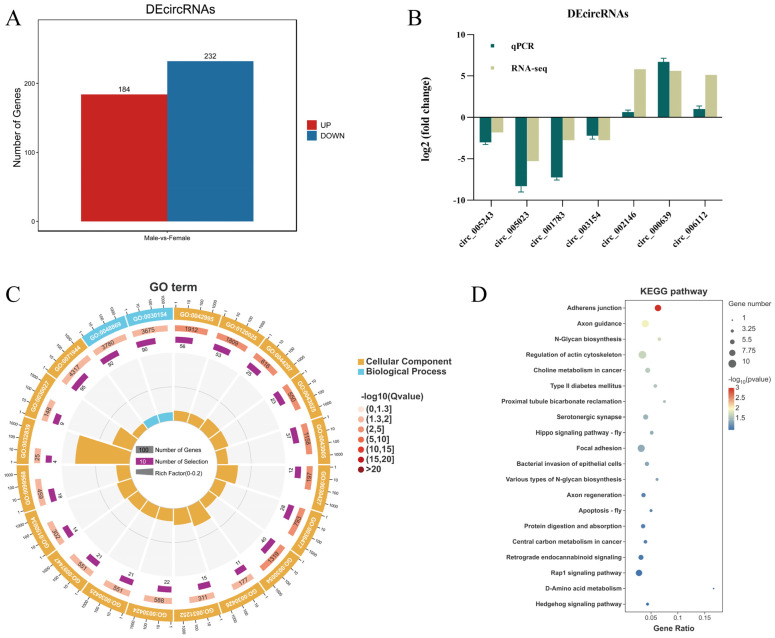
Analysis of circRNA profiles in male and female *M. armatus* gonads. (**A**) Identifying DEcircRNAs between male and female gonads. (**B**) The qPCR validation of seven randomly selected DEcircRNAs and qPCR data are presented as mean ± standard deviation. (**C**) GO enrichment analysis of genes encoding DEcircRNAs, exhibiting top 20 significantly enriched GO terms. (**D**) KEGG enrichment analysis of genes encoding DEcircRNAs, exhibiting top 20 enriched KEGG pathways.

Moreover, 416 differentially expressed circRNAs (DEcircRNAs) were also identified in the RNA-seq data, including 184 upregulated and 232 downregulated (Figure 4A) (Table S4). The reliability of the RNA-seq data was further supported by qPCR validation of seven randomly selected DEcircRNAs, whose expression patterns were in good agreement with the transcriptome data (Figure 4B). Functional enrichment analysis of the genes encoding DEcircRNAs found significant enrichment in 41 GO terms and 12 KEGG pathways (Figure 4C,D) (Table S4), with the Hippo signaling pathway among those associated with sex differentiation.

### 2.5. Identification and Characterization of Differentially Expressed miRNAs

**Figure 5:**
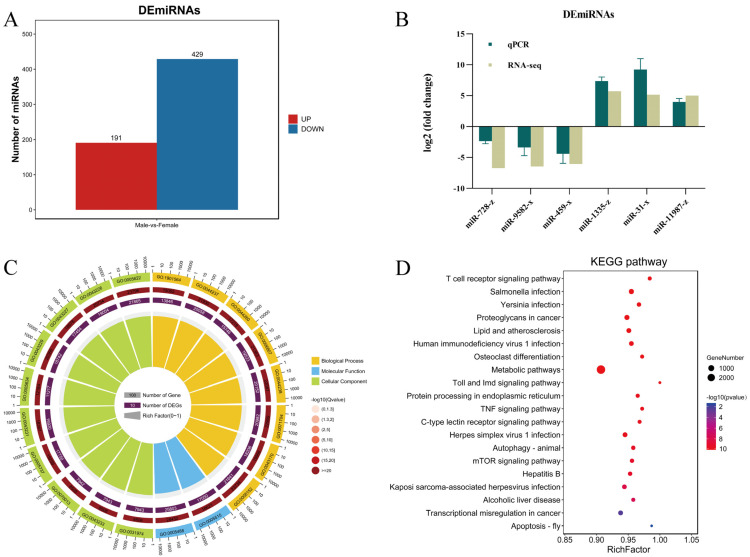
Analysis of miRNA profiles in male and female *M. armatus* gonads. (**A**) Identifying DEmiRNAs between male and female gonads. (**B**) The qPCR validation of six randomly selected DEmiRNAs and qPCR data are presented as mean ± standard deviation. (**C**) GO enrichment analysis of the target genes of DEmiRNAs, presenting top 20 significantly enriched GO terms. (**D**) KEGG enrichment analysis of the target genes of DEmiRNAs, presenting top 20 significantly enriched KEGG pathways.

Additionally, the RNA-seq of miRNA in the gonads of male and female *M. armatus* was conducted, identifying a total of 620 differentially expressed miRNAs (DEmiRNAs), with 191 upregulated and 429 downregulated (Figure 5A) (Table S5). The qPCR validation of six randomly selected DEmiRNAs gave results that agreed well with the transcriptome data (Figure 5B). Functional enrichment analysis of the target genes of DEmiRNAs indicated significant enrichment in 1403 GO terms and 172 KEGG pathways (Figure 5C,D) (Table S5). Among these, terms and pathways associated with sex differentiation included the Wnt signaling pathway, the Hedgehog signaling pathway, the MAPK signaling pathway, and so on. The basic information and PCA clustering plot for miRNA sequencing are presented in Table S6 and Figure S2, respectively.

### 2.6. Construction of the lncRNA/circRNA–miRNA–mRNA Regulation Networks

**Figure 6:**
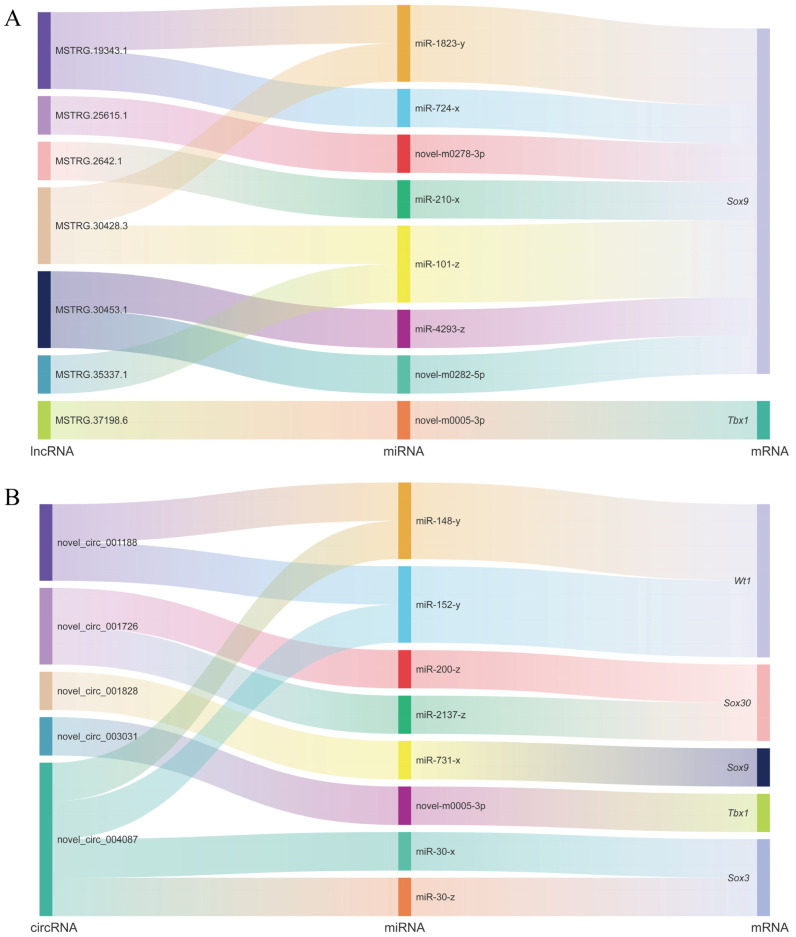
Construction of the interaction network of lncRNA–miRNA–mRNA (**A**) and circRNA–miRNA–mRNA (**B**) for candidate genes involved in sex differentiation of *M. armatus*. Different colored rectangular blocks in the Sankey plot represent individual RNA molecules, with color bands between blocks indicating potential regulatory relationships and the width of each band representing the strength of the correlation coefficient for that regulatory relationship.

Construction of the lncRNA/circRNA–miRNA–mRNA regulatory networks (Table S7) for candidate genes involved in sex differentiation of *M. armatus* revealed that *Sox9* is regulated by seven miRNAs and six lncRNAs, while *Tbx1* is regulated by one miRNA and one lncRNA within the lncRNA–miRNA–mRNA network (Figure 6A). Simultaneously, the circRNA–miRNA–mRNA regulatory network demonstrated that *Wt1* is regulated by two miRNAs and two circRNAs, whereas *Sox30* and *Sox3* are regulated by two miRNAs and one circRNA, and *Sox9* and *Tbx1* are regulated by one miRNA and one circRNA (Figure 6B).

## 3. Discussion

In this study, we systematically characterized the sexually dimorphic expression profiles of mRNAs, lncRNAs, circRNAs, and miRNAs in the gonads of *M. armatus*, a species exhibiting significant male-biased growth superiority [22,23]. Our integrated transcriptomic analysis not only confirmed the differential expression of key sex differentiation-related genes but also revealed complex non-coding RNA-mediated regulatory networks, providing a comprehensive molecular framework for understanding sex differentiation and sexually dimorphic growth in this economically important fish. The observed sexual dimorphism in body weight and multiple linear measurements align with previous reports in *M. armatus* [24,25], reinforcing the species’ potential for all-male aquaculture. These phenotypic differences are likely underpinned by the extensive transcriptomic divergence observed between male and female gonads, where thousands of DEmRNAs, DElncRNAs, DEcircRNAs, and DEmiRNAs were identified.

Among DEmRNAs, a large number of male-biased genes like *Sox30*, *Tbx1*, *Sox9*, and *Gata4* and female-biased genes like *Sox3*, *Foxl2*, and *Wnt4a* have been identified. *Sox30* is a key regulator of male germ cell differentiation [26] and is under the cis-regulation of the male sex-determining gene *Dmrt1* in *Nile tilapia* [27]. In female-to-male sex reversal individuals, the expression pattern of the *Tbx1* gene resembled that of normal males, and knockdown of *Tbx1* leads to upregulation of the female marker genes *Cyp19a* and *Foxl2* and downregulation of the male marker genes *Dmrt1* and *Amh* [28]. *Sox9* is a hub gene in male gonadal development and a target gene of the mammalian male sex-determining gene *Sry* [29]. *Gata4* targets the *Sox9* gene promoter to enhance its enhancer activity, proving crucial for male sex differentiation in mice [30]. *Sox3* is involved in female sex determination in *Glandirana rugosa* and knockdown of *Sox3* causes sex reversal from female to male [31]. *Foxl2* drives the development of coelomic epithelial cells into early supporting gonadal-like cells during early ovarian development, thereby maintaining the activation of the feminization pathway [32]. Rspo1/Wnt4/Ctnnb1 signaling strictly regulates the differentiation process of the ovary [33]. The upregulation of female-biased genes and the downregulation of male-biased genes reflect the conserved antagonistic regulation of ovarian and testicular pathways [34]. Functional enrichment analysis found that differentially expressed genes were enriched in pathways related to sex differentiation, such as the PI3K-Akt signaling pathway, the estrogen signaling pathway, and the TGF-beta signaling pathway. Knockout of *Igf3* leads to a reduction in germ cells in the early ovary by inhibiting the PI3K/Akt signaling pathway, thereby causing a reversal of the phenotype [35]. Estrogen signaling pathway is a highly conserved core regulatory module in the process of sex differentiation in vertebrates, which mediates downstream signaling cascades through estrogen receptors and plays a critical role in maintaining ovarian fate and development [36]. The TGF-beta signaling pathway is the primary signaling pathway involved in sex determination in teleosts and the sex-determining genes in 72 species have been confirmed to belong to the TGF-beta superfamily [37].

Meanwhile, previous studies have shown that ncRNAs are extensively involved in the process of sex differentiation [15]. Likewise, we also identified abundant differentially expressed lncRNAs, circRNAs, and miRNAs in both female and male gonads of *M. armatus*. Enrichment analysis of their regulatory genes, encoding genes, and target genes revealed that pathways associated with sex differentiation, including the GnRH secretion, the cortisol synthesis and secretion, the steroid hormone biosynthesis, the Hippo signaling pathway, the Wnt signaling pathway, the Hedgehog signaling pathway, and the MAPK signaling pathway, were significantly enriched. Gonadotropin-releasing hormone (GnRH) is synthesized and secreted in the hypothalamus and stimulates the release of gonadotropins, thereby promoting the differentiation of the ovaries and testes [38]. Cortisol is a hormone directly associated with stress, which induces a shift in the direction of sex differentiation by triggering steroid production during temperature-induced and socially regulated sex changes [39]. Estrogen and androgen are two key hormones involved in sex differentiation within the pathway of the steroid hormone biosynthesis, and treatment with estrogen and androgen prior to sex differentiation can reverse male and female genders, respectively [36]. *Yap* and *Wwtr1* are downstream effectors of the Hippo signaling pathway, and simultaneous knockout of these two genes induces a decrease in the mRNA levels of the male differentiation genes *Dmrt1* and *Sox9* in supporting cells, while the mRNA levels of the female differentiation genes *Rspo1* and *Foxl2* increase [40]. *Rspo1* and *Wnt4* cooperate to activate ovarian development in the bipotent gonad via the canonical Wnt signaling pathway [41]. Cholesterol promotes male sex differentiation through its roles in androgen synthesis and the transduction of the Hedgehog signaling pathway [42]. *Gnrh3* regulates the proliferation of primordial germ cells via the MAPK-dependent pathways and plays a crucial role in early sexual differentiation [38].

Additionally, miRNAs can target homologous mRNA sequences to induce the degradation of target genes, whereas lncRNAs or circRNAs act like sponges to bind miRNAs, thereby releasing mRNAs and restoring their expression levels [43]. Consequently, the ceRNA regulatory network constructed in this study provides a mechanistic hypothesis for understanding how ncRNAs coordinate sexual differentiation. In the regulatory network, genes associated with sex differentiation are regulated by one or multiple pairs of lncRNA/circRNA-miRNA. Previous studies have also found that the deletion of the miRNA cluster miR-17~92 leads to delayed *Sry* expression in male mice, impaired Sertoli cell differentiation, and an inability to sustain testis development, thereby inducing a sex reversal from male to female [44]. CircRNA *circdmrt1* and lncRNA *AMSDT* exhibit significantly high expression in the testis of *Cynoglossus semilaevis*, where they bind to cse-miR-196 to upregulate *gsdf* expression while downregulating the expression of female-specific genes, thereby promoting male sex differentiation [20]. *circSnd1* combines with mal-miR-135b and mal-miR-135c to regulate the expression of *Foxl2*, thereby participating in the process of sex differentiation in *Monopterus albus* [45]. Consequently, these predicted lncRNA/circRNA–miRNA–mRNA regulatory networks may represent a potential mechanism underlying sex differentiation of *M. armatus*, though this requires further validation through in vivo and in vitro functional experiments.

## 4. Materials and Methods

### 4.1. Sample Collection and Measurement

A total of 18 adult *M. armatus* was collected, comprising nine females and nine males, from the Huizhou Wealth Xing Industrial Co., Ltd. (Huizhou, Guangdong, China). Body weight was measured using an electronic balance (LKDZ3001, Beijing, China), while body length, body height, body width, head length, snout length, eye diameter, and interocular distance were measured using a vernier caliper (MNT, Shanghai, China). The morphological parameters were measured according to Figure S3. The Kolmogorov–Smirnov test and Levene’s test were used to assess the normality of the distribution and the homogeneity of variance of the morphological data for male and female individuals, respectively. A *t*-test was performed using SPSS Statistics 26 to analyze the significance of morphological parameters between males and females, with a *p*-value of less than 0.05 considered statistically significant.

### 4.2. Gonadal Tissue Sampling and Total RNA Isolation

After measurement, the fish were anesthetized and then humanely sacrificed. The sex of *M. armatus* was confirmed through anatomical examination (Figure S4). Subsequently, gonadal tissues were collected from both males and females, with three individuals pooled into each tube to ensure three biological replicates. The samples were immediately stored in liquid nitrogen. Total RNA was extracted from all samples using TRIzol reagent (Ambion, Carlsbad, CA, USA), with the process following the manufacturer’s instructions. The concentration and quality of total RNA were detected using NanoQ^TM^ (Thermo Scientific, Madison, WI, USA) and Agilent 2100 bioanalyzer (Agilent Technologies, Santa Clara, CA, USA), respectively.

### 4.3. Library Construction and Sequencing

Removing rRNA from high-quality RNA using the Ribo-Zero™ rRNA Removal kit (Epicenter, Madison, WI, USA) to preserve mRNA and non-coding RNA. Fragmented RNA templates were synthesized into the second-strand cDNA, followed by end repair, adenylation, and ligation of adapters. The ligation products were purified, and the target fragments were selected by agarose gel electrophoresis, followed by PCR amplification and sequencing using Illumina HiSeqTM 4000 by Gene Denovo Biotechnology Co. (Guangzhou, China). For miRNA, the SE150 (single-end 150 bp) strategy was employed for library preparation and sequencing. Concretely, the RNAs within the size range of 18–30 nt were enriched through polyacrylamide gel electrophoresis. Subsequently, ligation of adapters, reverse transcription, and PCR amplification were performed. The 140–160 bp size PCR products were enriched to generate cDNA libraries and sequenced using Illumina HiSeq Xten by Gene Denovo Biotechnology Co. (Guangzhou, China).

### 4.4. Identification of Differentially Expressed mRNAs and Non-Coding RNAs

Raw data underwent quality control using fastp v1.3.4 [46], and clean reads were aligned to the reference genome of *M. armatus* [47] using HISAT2 2.1.0 [48], with mapped reads assembled using Stringtie v1.3.1 [49]. LncRNAs were identified using CPC2 v1.0.1 [50], CNCI v2 [51], and FEELnc v0.2.1 [52] software, while circRNAs were identified using CIRIQuant v1.1.3 [53]. The miRNA identification was based on alignment against the miRBase database [54] and prediction using the miRDeep2 software v0.1.3 [55]. The fragments per kilobase per million reads (FPKM) and the transcripts per kilobase of exon model per million mapped reads (TPM) values were calculated using RSEM v1.3.3 [56]. Differentially expressed mRNAs and ncRNAs were evaluated using DESeq2 v1.4 [57], with thresholds set at the false discovery rate (FDR) < 0.05 and the absolute fold change ≥ 2.

### 4.5. Functional Enrichment Analysis

LncRNAs can regulate gene transcription through antisense, cis-acting, and trans-acting mechanisms [21]. Therefore, GO and KEGG enrichment analyses were performed on the genes affected by DElncRNAs, the identified DEmRNAs, the genes encoding DEcircRNAs, and the genes targeted by DEmiRNAs. GO terms with *q*-values below 0.05 and KEGG pathways with *p*-values below 0.05 were considered significantly enriched.

### 4.6. Construction of the ceRNA Network

To better understand the role of ncRNAs in regulating mRNA transcription during sex differentiation in *M. armatus*, a ceRNA network involving lncRNA/circRNA–miRNA–mRNA was constructed. Concretely, the expression correlation between mRNA–miRNA or lncRNA–miRNA was evaluated using the Spearman Rank correlation coefficient (SCC). Pairs with SCC < −0.7 were selected as co-expressed negatively lncRNA–miRNA pairs or mRNA–miRNA pairs, both mRNA and lncRNA were miRNA target genes, and all RNAs were differentially expressed. Expression correlation between lncRNA–mRNA was evaluated using the Pearson correlation coefficient (PCC). Pairs with PCC > 0.9 were selected as co-expressed lncRNA–mRNA pairs, both mRNA and lncRNA in this pair were targeted and co-expressed negatively with a common miRNA. The hypergeometric cumulative distribution function test to verify whether the common miRNA sponges between the two genes were significant. As a result, only the gene pairs with a *p*-value less than 0.05 were selected. The lncRNA/circRNA–miRNA–mRNA network sex differentiation genes was constructed by assembling all co-expression competing triplets, which were identified above, and was visualized using Cytoscape software v3.6.0 (http://www.cytoscape.org/).

### 4.7. Validation of Transcriptome Data

To validate the accuracy of the transcriptomic data, several DEmRNAs, DElncRNAs, DEcircRNAs, and DEmiRNAs were randomly selected for qPCR analysis. The qPCR data for DEmRNAs, DElncRNAs, and DEcircRNAs were normalized using the *β-actin* gene [58], while DEmiRNAs were normalized using the *U6* gene [59]. The relative expression levels were calculated using the 2^−∆∆Ct^ method [60] and all primers used in the qPCR analysis are listed in Table S8.

## 5. Conclusions

This study reveals the complex molecular landscape of sexual dimorphism in the gonads of *M. armatus*, constructs a regulatory network centered on ceRNA, and identifies key genes and pathways involved in sex differentiation, thereby laying a molecular foundation for a deeper understanding of the mechanisms underlying sex differentiation and for the development of all-male breeding in *M. armatus*.

## Data Availability

Raw data can be downloaded from the Genome Sequence Archive at the National Center for Biotechnology Information (CRA042343).

## Supplementary Materials

The following supporting information can be downloaded at: https://www.mdpi.com/article/10.3390/ijms27115111/s1.
