# Supplementary figures and images for: Integrated Transcriptome Landscape of mRNAs, lncRNAs, circRNAs, and miRNAs Reveals Molecular Regulatory Networks of Sex Differentiation in the Zig-Zag Eel (Mastacembelus armatus)
Source: Int J Mol Sci. 2026 Jun 5;27(11):5111. doi: 10.3390/ijms27115111 (PMC13256884; doi:10.3390/ijms27115111)

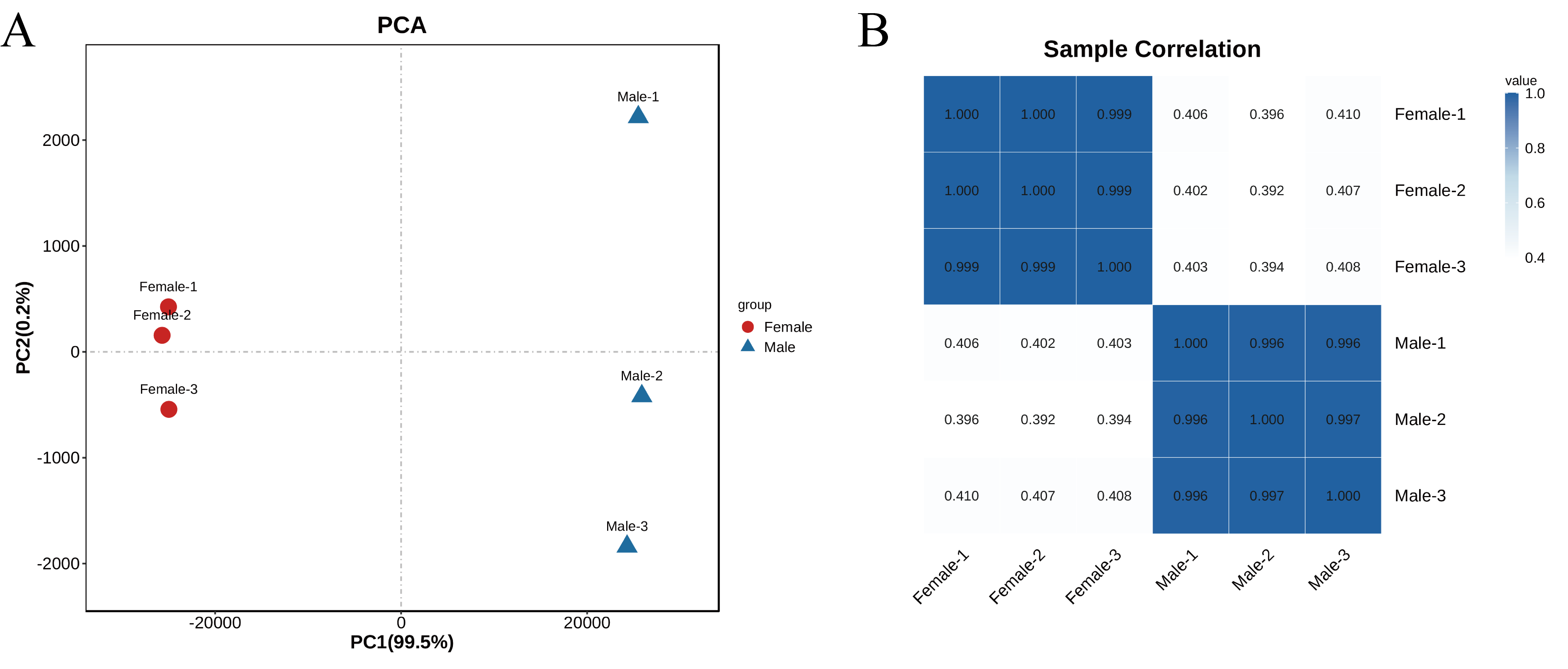

Supplement: Supplementary file 1 [file ijms-27-05111-s001.zip › Figure S1.Principal component analysis (A) and correlation analysis (B) of gonadal samples from male and female M. armatus.tif]

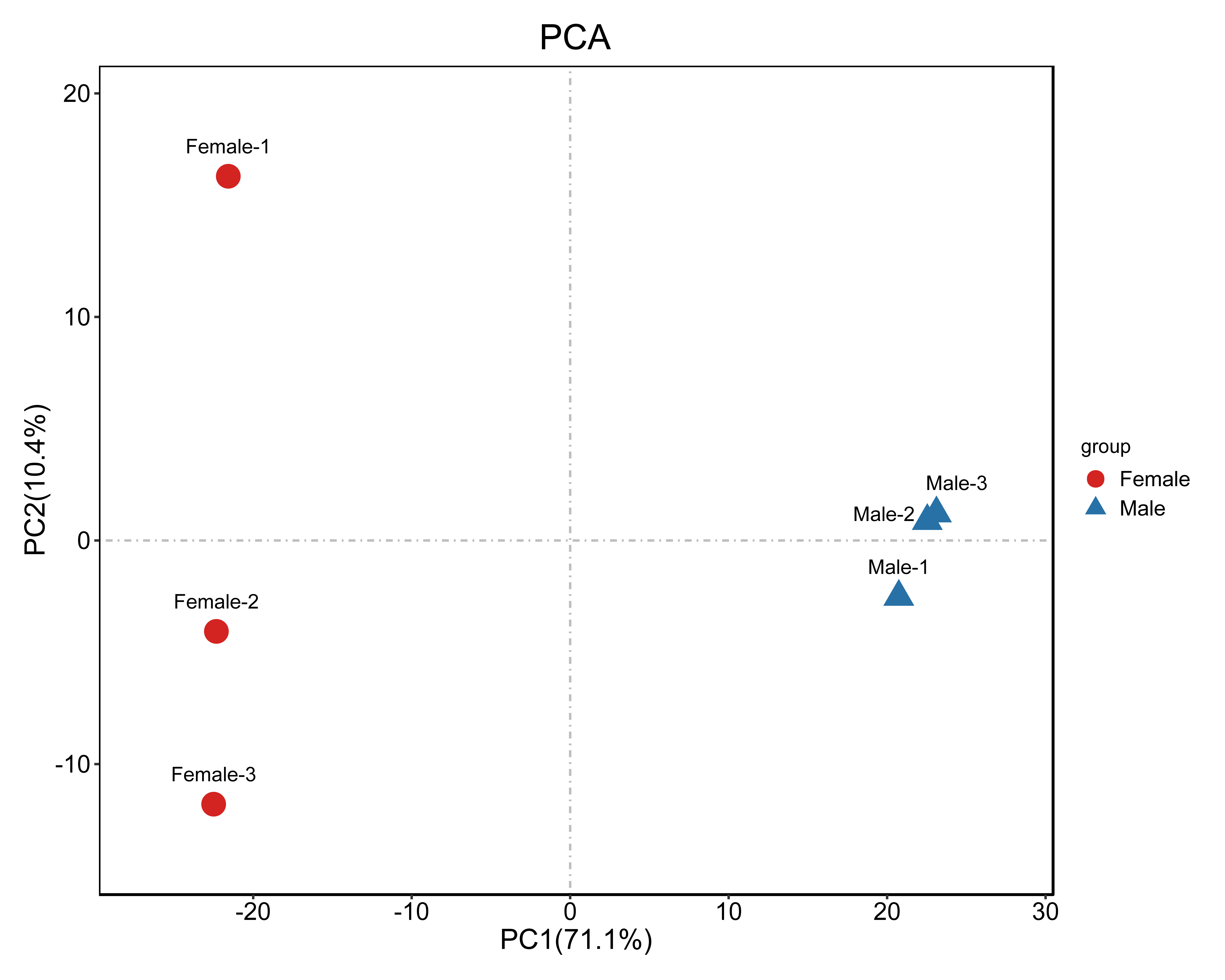

Supplement: Supplementary file 1 [file ijms-27-05111-s001.zip › Figure S2.Principal component analysis of miRNA sequencing samples.tif]

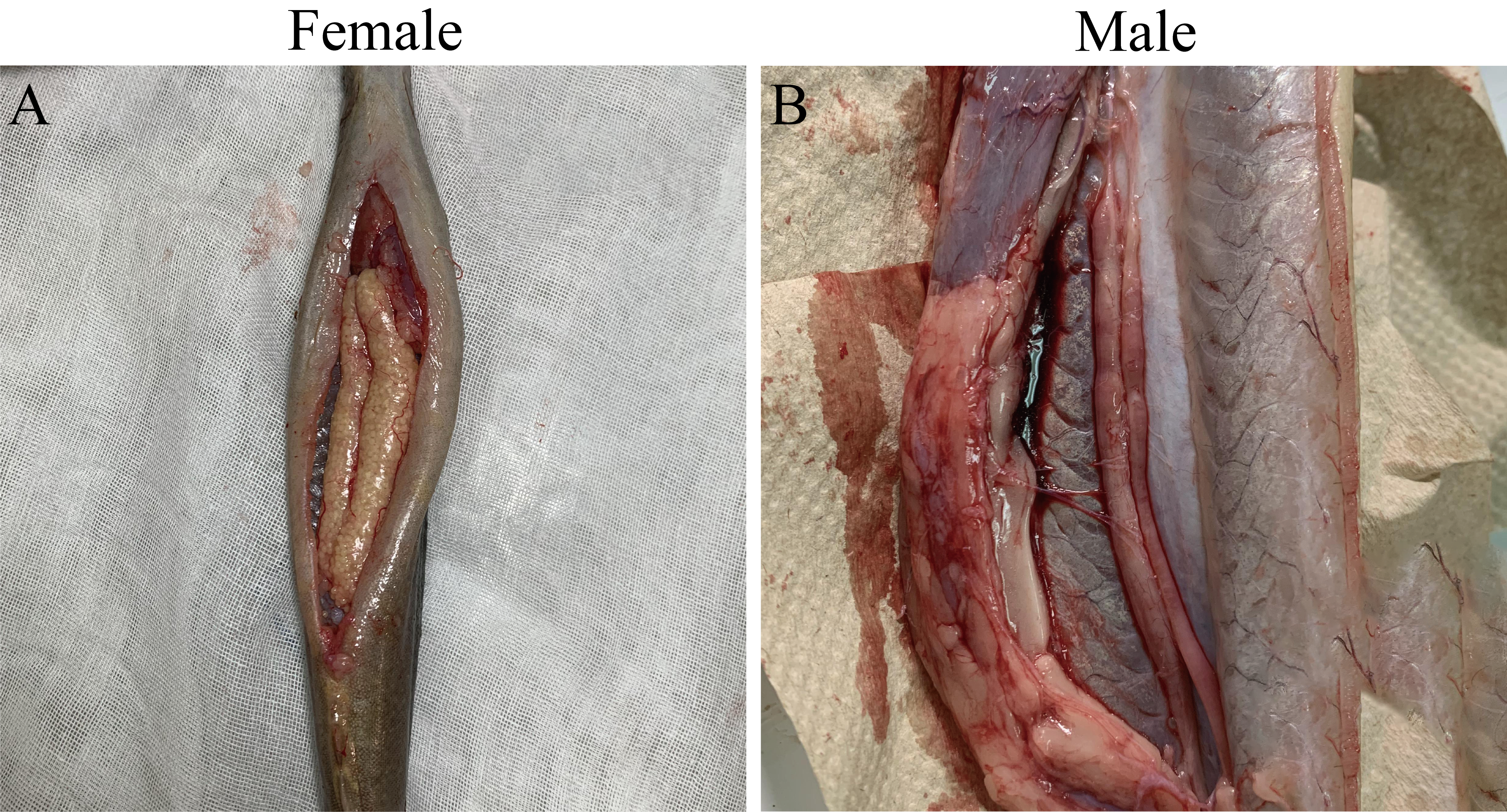

Supplement: Supplementary file 1 [file ijms-27-05111-s001.zip › Figure S4.Illustrative images of gonadal sampling in female (A) and male (B) M. armatus.tif]
